# NLRP3 inflammasome activation in murine macrophages caused by *Neospora caninum* infection

**DOI:** 10.1186/s13071-017-2197-2

**Published:** 2017-05-30

**Authors:** Xiaocen Wang, Pengtao Gong, Xu Zhang, Jielin Wang, Lixin Tai, Xu Wang, Zhengkai Wei, Yongjun Yang, Zhengtao Yang, Jianhua Li, Xichen Zhang

**Affiliations:** College of Veterinary Medicine, Jilin University, Jilin Changchun, 130062 China

**Keywords:** *Neospora caninum*, Macrophages, NLRP3 inflammasome, Caspase-1, IL-1β

## Abstract

**Background:**

*Neospora caninum* is an intracellular parasite that causes significant economic losses in cattle industry. Understanding the host resistance mechanisms in the innate immune response to neosporosis could facilitate the exploration of approaches for controlling *N. caninum* infection. The NLR inflammasome is a multiprotein platform in the cell cytoplasm and plays critical roles in the host response against microbes.

**Methods:**

*Neospora caninum*-infected wild-type (WT) macrophages and *Nlrp3* ^*−/−*^ macrophages, and inhibitory approaches were used to investigate inflammasome activation and its role in *N. caninum* infection. Inflammasome RT Profiler PCR Arrays were used to identify the primary genes involved in *N. caninum* infection. The expression of the sensor protein NLRP3, processing of caspase-1, secretion of IL-1β and cell death were detected. *Neospora caninum* replication in macrophages was also assessed.

**Results:**

Many NLR molecules participated in the recognition of *N. caninum*, especially the sensor protein NLRP3, and further study revealed that the NLRP3 distribution became punctate in the cell cytoplasm, which facilitated inflammasome activation. Inflammasome activation-mediated caspase-1 processing and IL-1β cleavage in response to *N. caninum* infection were observed and were correlated with the time of infection and number of infecting parasites. LDH-related cell death was also observed, and this death was regarded as beneficial for the clearance of *N. caninum.* Treatment of *N. caninum*-infected macrophages with caspase-1, pan-caspase and NLRP3 inhibitors led to the impaired release of active IL-1β and a failure to restrict parasite replication. And *Neospora caninum* infected peritoneal macrophages from *Nlrp3*-deficient mice displayed greatly decreased release of active IL-1β and the failure of caspase-1 cleavage.

**Conclusions:**

The NLRP3 inflammasome can be activated in *N. caninum*-infected macrophages, and plays a protective role in the host response to control *N. caninum*.

**Electronic supplementary material:**

The online version of this article (doi:10.1186/s13071-017-2197-2) contains supplementary material, which is available to authorized users.

## Background

*Neospora caninum* is a protozoan parasite with a wide host range. It mainly causes a serious disease in cattle and dogs worldwide [1]. Neosporosis, which leads to significant economic losses in both the dairy and beef industries around the world, is a major cause of abortions in the cattle industry, and has been associated with neonatal mortality and neurological clinical signs in congenitally infected calves [2–4]. Although similar in life-cycle, subcellular ultrastructure, invasion mechanism, metabolic pathway and genome organization, *N. caninum* differs from *Toxoplasma gondii* in host range, virulence factors and disease pathogenesis [5]. Analyses of the genome of *Neospora* and the transcriptomes of both *Neospora* and *Toxoplasma* have shown that there are many differences in the groups of genes that interact with the host, and these differences may lead to changes in interactions with the host immune mechanisms [6]. Therefore, a good understanding of the immune mechanisms that mediate host resistance to neosporosis may facilitate the discovery of approaches to control neosporosis.

As it is an intracellular parasite, the intracellular proliferation of *N. caninum* tachyzoites is a key step in the pathogenesis of neosporosis. The innate immune system is the first line of defense in host resistance to *N. caninum* infection, playing an important role in the control of the initial parasite replication and then mediating an appropriate adaptive immune response [7]. Pattern recognition receptors (PRRs) of innate immune cells, such as macrophages, can sense microbes by recognizing the pathogen-associated molecular patterns (PAMPs) of pathogens [8]. Studies show that TLR2 [9] and TLR3 [5], which belong to the Toll-like receptors (TLRs) family, participate in the initial recognition of *N. caninum*, induce the secretion of the pro-inflammatory cytokines IL-12 and IFN-γ, and mediate the immune response against *N. caninum*.

In recent years, studies have reported that another group of PRRs, termed nucleotide oligomerization domain (Nod)-like receptors (NLRs), plays important roles in the host response to the pathogenesis of infections caused by intracellular parasites [10]. NLRs are intracellular cytosolic sensors and are defined by a tripartite structure [11]. They can be divided to two groups. One group mediates NF-κB activation via NOD1 and NOD2 [12], and a recent *N. caninum* study shows that the murine NOD2-mediated response contributes to *N. caninum* elimination and the pathogenesis of neosporosis [7]. Another group of NLRs can sense multiplying PAMPs or danger-associated molecular patterns (DAMPs) and induce the assembly of the inflammasome, a multiprotein platform in the cell cytoplasm, to initiate the host defense against infectious pathogens [13]. The NLRP3 inflammasome senses various molecules, such as extracellular ATP, nigericin and uric acid crystals [14], and numerous bacteria, viruses, fungi and parasites [11]. Activation of the murine NLRP3 inflammasome requires two signals in macrophages. The first signal is typically provided by the activation of NF-κB (after LPS treatment, for example) and induces the transcriptional upregulation of NLRP3 and pro- IL-1β/pro- IL-18; the second signal is provided by the above stimuli and then causes inflammasome complex formation, and caspase-1 is engaged to proteolytically cleave pro-IL1β/pro-IL-18 into active IL-1β/IL-18 [11]. Meanwhile, active caspase-1 also triggers a form of programmed cell death known as pyroptosis by cleaving GSDMD [15]. Different from necroptosis and apoptosis, pyroptosis is a lytic form of cell death and is caused by the GSDMD-formed pores in the plasma membrane [16, 17], and this programmed cell death is also essential for immune defenses and diseases.

NLRP1 is responsible for the host susceptibility to *Toxoplasma* infection, and knockdown of the NLRP1 gene directly influences inflammasome related-cell death and parasite replication in *Toxoplasma*-infected rat macrophages [18]. NLRP3 and other inflammasome components also play protective roles in the host response to *T. gondii* infection [19]. However, the inflammasome responses in *N. caninum* infection are poorly understood. Therefore, this is the first study to focus on whether the inflammasome participates in innate immune recognition and its role in the host defense against *N. caninum*.

## Methods

### Animals

Female C57BL/6 mice (6–8 weeks old) were obtained from the Laboratory Animal Center of Jilin University (Changchun, China). *Nlrp3* ^*−/−*^ (knockout) mice were purchased from The Jackson Laboratory (Bar Harbor, ME, USA). The mice were maintained in isolator cages, with a light/dark cycle of 12 h, and with sterile food and water in the animal house of the Laboratory Animal Center of Jilin University.

### Parasites and cells

*Neospora caninum* tachyzoites (Nc-1) were maintained by serial passages in Vero cells, and free *N. caninum* were obtained from the cell cultures as described previously [20]. Briefly, *N. caninum* tachyzoites were harvested after 80% lysis of the host cells by mechanical disruption with a 27 gauge needle and centrifuged at 1500× *g* for 30 min to remove host cell debris by gradient density centrifugation with a 40% Percoll (GE Healthcare, Uppsala, Sweden) solution (v/v). The pellet of the parasite suspension was collected and washed twice with RPMI-1640 (centrifuged at 1000× *g* for 10 min), and the *N. caninum* concentration was determined in a hemocytometer. Excretory secretory antigens (ESAs) of *N. caninum* tachyzoites were prepared, and 2 × 10^8^ tachyzoites were incubated at 37 °C for 3 h in 2 ml of serum-free RPMI-1640 (Life Technologies, Grand Island, NY) containing penicillin-streptomycin. After centrifugation for 10 min at 1000× *g*, the ESAs-containing supernatant was filtered through a 0.22-μm Millipore membrane and stored at -80 °C until use [21].

The mice were inoculated intraperitoneally with 3 ml of 5% thioglycollate medium for 4 days, and macrophages from the peritoneal cavity were harvested in cold PBS [22]. Peritoneal macrophages (PMs) were cultured in complete medium (RPMI, 2 mM glutamine, 1 mM sodium pyruvate, 10 mM HEPES and 100 μg penicillin-streptomycin) plus 10% fetal bovine serum (FBS; BI, Shanghai, China) for 24 h in 6-well plates at 1 × 10^6^ cells/well.

### Probing the mouse inflammasome RT profiler PCR arrays

PMs were infected with *N. caninum* at a multiplicity of infection (MOI) of 3:1 (parasite:cell) for 12 h and 24 h and harvested with 1 ml of TRIzol reagent (Life Technologies, Carlsbad, USA) for total RNA extraction according to the manufacturer’s instructions. After quantification using a Nanodrop ND-2000 apparatus (Thermo Scientific, Wilmington, USA), 2 μg of total RNA was used for cDNA synthesis using the RT^2^ First Strand Kit (SA Biosciences/Qiagen, Shanghai, China).

The 96-well Mouse Inflammasome RT Profiler PCR Arrays (SA Biosciences/Qiagen, Shanghai, China), containing primer pairs for 84 key genes involved in the NLR inflammasome pathway, were probed with cDNA template in the presence of SuperArray PCR master mix (Qiagen, Shanghai, China), and the PCR analysis was performed on a Bio-Rad iCycler (USA). The arrays were probed with cDNA from 3 treatments (12 h, 24 h and the control group) in duplicate, and the results were analyzed by Excel-based PCR Array Data Analysis software (SA Biosciences, Shanghai, China) for threshold cycle (Ct) value determination. The Ct of the genes was normalized to that of housekeeping genes (the average Ct of Actb, B2m, Gapdh and Hsp90ab1). The relative expression level of each target gene in infected cells was calculated as 2^-ΔΔCt^ (fold change), where ΔΔCt represents the Ct (sample) - Ct (control).

### RNA isolation and real-time PCR analysis

Total RNA was extracted from infected peritoneal macrophages (MOI = 3:1) at 12 h post infection (p.i.) using TRIzol reagent according to the manufacturer’s instructions. All RNA samples were dissolved in 20 μl of nuclease-free H_2_O and quantified using a Nanodrop ND-2000 apparatus (Thermo Scientific). cDNA synthesis was performed using 2–3 μg of total RNA prepared as described above in a 20 μl final volume using a PrimeScript™ RT Reagent Kit (TaKaRa, Dalian, China) according to the manufacturer’s instructions. Real-time PCR was then used for the quantification of murine NLRP3 [23], NLRC4 and NLRC5 (each specific primer is listed in Table 1) mRNA expression levels with FastStart Universal SYBR Green Master (Roche Diagnostics, Mannheim, Germany). The qPCR was performed as follows: denaturation at 95 °C for 5 min, followed by amplification with 40 cycles of 95 °C for 10 s and 60 °C for 30 s. The data were normalized to murine β-actin, and the fold change was calculated as 2^-ΔΔCt^, where ΔΔCt represents the Ct (sample) - Ct (control).

**Table 1:** Primer sequences used in this study

Gene	Accession number	Primer sequence (5'-3')	Size (bp)	Reference
Murine				
NLRP3	NM_145827	Forward: AGAAGAGACCACGGCAGAAG	102	[23]
		Reverse: CCTTGGACCAGGTTCAGTGT		
NLRC4	NM_001033367.3	Forward: CTTGGCCAGGAGAGCCTTG	153	
		Reverse: GGGCTCGTCTGTTGTTCCTT		
NLRC5	NM_001033207.3	Forward: GCTGAGAGCATCCGACTGAA	157	
		Reverse: GGTGGATGACCTCAGGGTTG		
ACTB	NM_007393.5	Forward: ACCTTCTACAATGAGCTGCG	147	
		Reverse: CTGGATGGCTACGTACATGG		
*Neospora caninum*				
NC5	X84238	Forward: ACTGGAGGCACGCTGAACAC	76	[25]
		Reverse: AACAATGCTTCGCAAGAGGAA		

### Stimulation

At 3 h prior to infection, the medium was changed to complete medium (RPMI) plus 1% fetal bovine serum with or without 100 ng/ml ultrapure lipopolysaccharide (LPS; Sigma, Shanghai, China). The PMs were stimulated with *N. caninum* for the indicated times (1–24 h) at various multiplicities of infection (MOI = 1:1, 3:1, and 5:1). PMs treated with medium alone or LPS were used as negative controls, and cells treated with ATP (5 mM, 30 min; Sigma, Shanghai, China) were used as positive controls, as extracellular ATP is a conventional NLRP3 inflammasome agonist [14].

To monitor the role of the inflammasome in response to *N. caninum* infection in PMs, PMs were pre-treated with 100 μM Ac-YVAD-CHO (an inhibitor of caspase-1 and -4; Enzo Life Science, Lausen, Switzerland), 100 μM zVAD-fmk (an inhibitor of pan-caspase; Selleck, Shanghai, China) or 100 μM glyburide (an inhibitor of NLRP3 by inhibiting K^+^ efflux; Selleck, Shanghai, China) for 45 min before stimulation, and the PMs were then stimulated with LPS priming plus *N. caninum* for 12 h at an MOI of 3:1 (parasite:cell), and PMs cultured with 0.2% DMSO were used as a negative control.

### Cytokine measurement and LDH assay

Supernatants were harvested at various time points for cytokine measurement and in select experiments for the lactate dehydrogenase (LDH) assay (Roche Diagnostics, Mannheim, Germany) according to the manufacturer’s protocol. The percentage of LDH release, as a measure of cell death, was calculated as follows: (LDH infected - LDH control)/(LDH total lysis - LDH control) × 100. The remaining supernatants were stored at -20 °C for use in an IL-1β enzyme-linked immunosorbent assay (ELISA) by a mouse IL-1β Ready-Set-Go Kit (eBioscience, San Diego, USA).

### Immunoblot analysis

Cell lysates were extracted from infected macrophages in radioimmunoprecipitation assay (RIPA) buffer with complete protease inhibitor, phenylmethylsulfonyl fluoride (PMSF) and phosphatase inhibitor cocktail (all from Sigma). Cell culture supernatants were precipitated by methanol and chloroform to obtain protein samples as previously described [24]. Briefly, the supernatants were mixed with an equal volume of cold methanol and 1/4 volume of cold chloroform and then centrifuged at 13,000× *g* for 10 min at 4 °C. The upper phase was discarded, 1 ml of methanol was added, and the mixture was then centrifuged for 10 min at 13,000× *g*. The protein pellets were dried at room temperature and dissolved in 1% SDS buffer. The protein samples were separated and analyzed by SDS-PAGE and immunoblot. Membranes were blocked in 5% skim milk, incubated overnight at 4 °C with primary antibodies and visualized with secondary HRP-conjugated antibodies (Proteintech, Wuhan, China). The primary antibodies used were anti-mouse IL-1β (AF-401, R&D, Minneapolis, USA), anti-mouse caspase-1 (p20) (AG-20B-0042, Adipogen, Liestal, Switzerland), anti-NLRP3 (AG-20B-0014, Adipogen, Liestal, Switzerland) and anti-mouse β-actin (60008–1, Proteintech, Wuhan, China).

### DNA extraction and detection of *N. caninum* by qPCR

The parasite replication in the infected cells was monitored as previously described [25] by performing a quantitative real-time PCR (qPCR) analysis of the parasite DNA. Genomic DNA from 10^7^ tachyzoites of *N. caninum* and total DNA from infected cells were extracted using a Genomic DNA Extraction Kit (TIANGEN, Beijing, China) according to the manufacturer’s protocol. The total DNA (100 ng) from infected cells was used as a template in qPCR analyses with FastStart Universal SYBR Green Master, and oligonucleotide primers specific for the Nc5 sequence of *N. caninum* (Table 1) were used to amplify a 76-bp DNA fragment. The parasite number was determined by a standard curve performed with DNA isolated from *N. caninum* tachyzoites, ranging from 5 to 5 × 10^5^ parasites, included in each run. The data were analyzed and expressed as log10 parasites per 100 ng of total DNA.

### Confocal microscopy

PMs were plated on coverslips in 6-well plates, pre-treated with LPS (100 ng/ml) for 3 h, and then stimulated with *N. caninum* tachyzoites for 10 h (MOI = 3). After washing two times with PBS, the cells were fixed with 4% paraformaldehyde in PBS for 15 min and then washed three times with PBS. After a 20-min permeabilization with 0.5% Triton X-100 in PBS and blocking with 3% BSA in PBS for 30 min at room temperature, the cells were incubated with an antibody against NLRP3 (Adipogen; 1:200) overnight at 4 °C. After washing with PBST, the cells were incubated with secondary antibody (Proteintech) for 1 h at room temperature. F-actin was stained with TRITC Phalloidin, and mammalian nuclei were stained with DAPI. For visualization of live or dead infected cells, the PMs were seeded in 96-well plates, and after stimulation for 12 h the cells were stained using a LIVE/DEAD Cell Imaging Kit (Life Technologies, Eugene, USA) for 15 min. The cells were analyzed on an Olympus FV1000 Laser Scanning Confocal microscope (Japan) with a 100× objective for NLRP3 imaging and 10× objective for live/dead cell imaging.

### Statistics

Data analysis was performed using Prism 5.0 (GraphPad Software, Inc.) and expressed as the mean ± SD. To evaluate the differences between two groups (experimental group *vs* control group), the two-tailed *t*-test was used. Significance is shown by *P < 0.05, **P < 0.01, ***P <0.001.

## Results

### Inflammasome-related gene expression in *N. caninum*-infected peritoneal macrophages

**Fig. 1:**
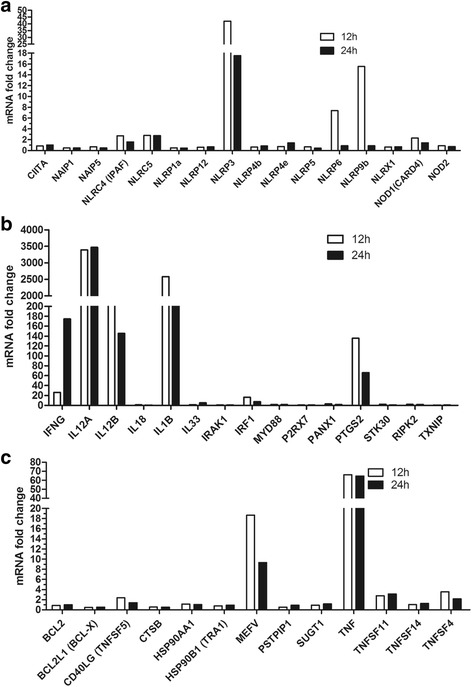
Inflammasome-related gene expression in mouse peritoneal macrophages in response to *N. caninum* infection. The 96-well RT Profiler Mouse Inflammasome PCR Arrays (Qiagen) were probed with cDNA from peritoneal macrophages infected with *N. caninum* (MOI = 3:1, parasite:cell) for 12 h or 24 h (without LPS priming). The Ct values from the qPCR data were analyzed, and the relative expression level of each target gene in the samples was calculated using fold change. Fold-Change (2^-ΔΔCt^) is the normalized gene expression (2^-ΔCt^) in the Test Sample divided the normalized gene expression (2^-ΔCt^) in the Control Sample. In the PCR arrays, 84 inflammasome-related genes were measured, and the expression levels of key genes in three functional groups are shown. **a** Relative expression levels of 16 NOD-like receptor genes. **b** Relative expression levels of downstream signaling genes in inflammasome-mediated responses. **c** Relative expression levels of inflammasome negative regulation genes. The data are representative of two independent experiments

Murine peritoneal macrophages were incubated with *N. caninum* for 12 or 24 h and probed for the expression of 84 genes using the Mouse Inflammasome RT Profiler PCR Arrays. Of the 84 probed genes, 40 (32 upregulated and 8 downregulated) and 33 (24 upregulated and 9 downregulated) genes in *N. caninum*-infected PMs were differentially expressed at 12 and 24 h p.i., respectively, in comparison to the expression observed in the control cells. The expression of 16 NOD-like receptor (sensor protein) genes were especially noted, and the results exhibited that 8 (6 upregulated and 2 downregulated) and 6 (2 upregulated and 4 downregulated) of these genes showed differential expression at 12 and 24 h p.i., respectively (Fig. 1a). The NLRP3 (41.90 ± 4.63, 12 h; 17.56 ± 2.45, 24 h) and NLRC5 (2.8 ± 0.47, 12 h; 2.73 ± 0.75, 24 h) genes were upregulated at both 12 and 24 h p.i. In addition, the NAIP1 (0.46 ± 0.12, 12 h; 0.48 ± 0.09, 24 h) and NLRP1a (0.46 ± 0.08, 12 h; 0.48 ± 0.13, 24 h) genes were downregulated at both 12 and 24 h p.i. Meanwhile, the expression levels of downstream signaling genes in the inflammasome-mediated response to *N. caninum* infection showed that IL-1B (IL-1β), IFNG (IFN-γ), IL-12A (IL-12p35/a), and IL-12B (IL-12p40) were extremely upregulated (Fig. 1b), while upregulation of IL-18 was not observed. The expression levels of inflammasome negative regulation genes showed that the expression of 6 (5 upregulated and 1 downregulated) and 5 (4 upregulated and 1 downregulated) genes was changed at 12 and 24 h p.i., respectively. The MEFV (FMF/TRIM20), TNF (TNF-α), TNFSF11 (Ly109l/ODF), and TNFSF4 (Ath1/CD134L) genes were upregulated at both 12 and 24 h p.i. (Fig. 1c). These results indicate that many types of genes in the inflammasome pathway participated in the response to *N. caninum* infection, several receptor genes are upregulated, and NLRP3 is the most upregulated sensor protein.

### Secretion of inflammasome-mediated IL-1β in *N. caninum*-infected peritoneal macrophages

**Fig. 2:**
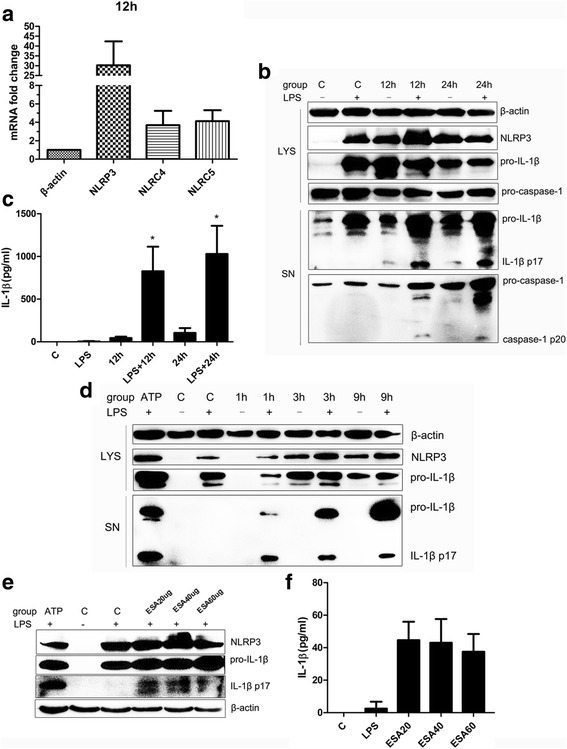
Secretion of inflammasome-mediated IL-1β is induced in mouse peritoneal macrophages infected by *N. caninum*. **a** Confirmation of some upregulated genes indicated in the inflammasome PCR array. Peritoneal macrophages (without LPS priming) were stimulated with *N. caninum* tachyzoites (MOI = 3:1, parasite:cell) for 12 h, and the expression levels of the NLRP3, NLRC4 and NLRC5 genes were measured by qPCR. **b**-**f** With or without LPS priming (indicated + or – in the figures), peritoneal macrophages were incubated with *N. caninum* tachyzoites (MOI = 3:1, parasite:cell) for the indicated time or *N. caninum* tachyzoites excretory-secretory antigens (ESAs) for 12 h; LPS primed peritoneal macrophages were incubated with ATP (5 mM) for 30 min as positive control. **b**, **d**, **e** Expression of NLRP3, processing of caspase-1 and secretion of IL-β were analyzed by immunoblot. **c**, **f** IL-1β production in supernatants was measured by ELISA. *Abbreviations*: C, control; SN, supernatants; LYS, cell lysates; ESA, excretory secretory antigens. The data are representatives of three independent experiments and presented as the mean ± SD (*P < 0.05; **P < 0.01; ***P < 0.001 *vs* negative control)

To verify the results regarding upregulated receptor genes (12 h p.i.) obtained in the PCR array analysis (Fig. 1a), the expression levels of NLRP3, NLRC4 and NLRC5 in *N. caninum-*infected PMs at 12 h p.i. were detected by qPCR (Fig. 2a). The qPCR results confirmed that NLRP3 was the most upregulated receptor. To examine inflammasome activation, the expression of NLRP3, processing and secretion of caspase-1, and production of the caspase-1 substrate IL-1β were examined in macrophages stimulated with *N. caninum*. In unstimulated macrophages, caspase-1 is a 45 kDa pro-enzyme. When recruited to an activated inflammasome platform, caspase-1 begins autoproteolysis to become the active form (p20 and p10 subunits) and cleaves pro-IL-1β (31 kDa) into active IL-1β (17 kDa) [26]. PMs with or without LPS priming were incubated with *N. caninum* (3:1, parasite:cell), and at 12 and 24 h p.i. the results showed a gradual increase in IL-1β production (Fig. 2b, c). In the absence of LPS priming, NLRP3 expression together with the secretion of active IL-1β p17 (bottom immunoblot) could be induced in *N. caninum*-infected PMs, and these data support the transcriptional upregulation of NLRP3 and IL-1β in Fig. 1, but it was difficult to detect active caspase-1 p20 (bottom immunoblot) (Fig. 2b). However, in the presence of LPS priming, more robust production of IL-1β and secretion of processed caspase-1 p20 subunits were observed (Fig. 2b). Moreover, in the presence of LPS priming, *N. caninum*-infected PMs released a substantially higher amount of IL-1β than that observed with only *N. caninum* infection, and in both the absence and presence of LPS priming, the production of IL-1β was gradually increased at 12 and 24 h p.i. in *N. caninum*-infected PMs (Fig. 2b, c). Although the expression of NLRP3 and pro-IL-1β can be observed, *N. caninum* infection was not sufficient to trigger the cleavage of IL-1β in PMs at 3 and 9 h p.i. in the absence of LPS priming (Fig. 2d). In contrast, in the presence of LPS priming, the cleavage of IL-1β can be observed as early as at 1 h p.i. in *N. caninum*-infected PMs, and LPS treatment alone could not induce cleavage of IL-1β in PMs (Fig. 2d). These data indicate that the processing and release of IL-1β induced in macrophages by *N. caninum* occurs in a time-dependent manner, and LPS priming is also essential for *N. caninum*-triggered inflammasome activation. In addition, IL-1β cleavage could be induced by treatment with excretory secretory antigens (ESAs) of *N. caninum* tachyzoites at 12 h p.i. in the presence of LPS priming (Fig. 2e, f).

### IL-1β production and cell death in *N. caninum*-infected peritoneal macrophages

**Fig. 3:**
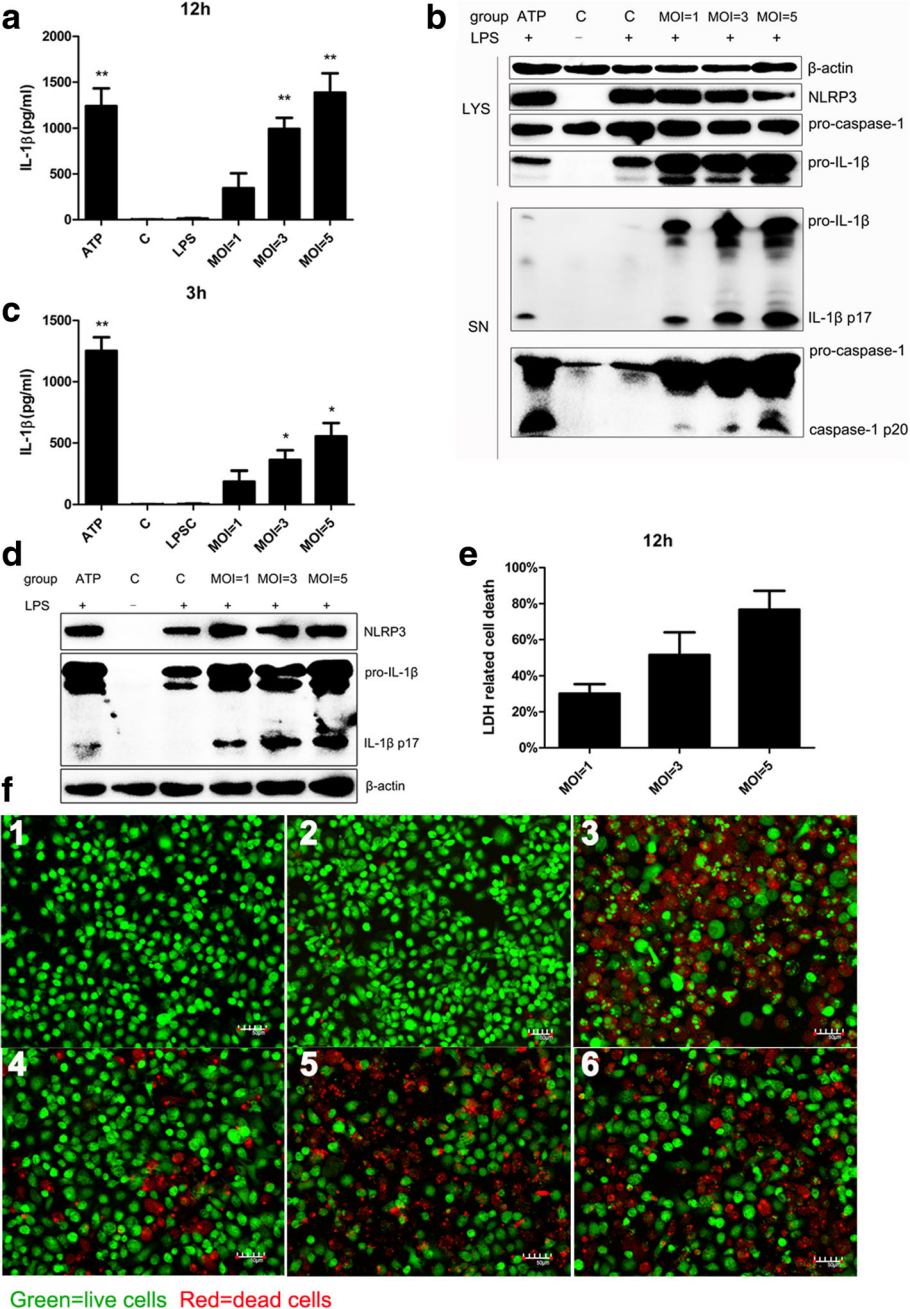
IL-1β production and cell death induced by *N. caninum* depends on the parasite amount in peritoneal macrophages. Peritoneal macrophages primed with LPS were incubated with the indicated number (MOI = parasite:cell) of *N. caninum* tachyzoites for 12 h (**a**, **b**, **e**, **f**) or 3 h (**c**, **d**), or incubated with ATP (5 mM) for 30 min as positive control. **a**, **c** The production of IL-1β in the supernatants was measured by ELISA. **b**, **d** The secretion of the processed p20 subunit and active IL-1β (p17) and the expression of NLRP3 were detected in cell extracts (LYS) or supernatants (SN) by immunoblot. **e** Supernatants were harvested at 12 h p.i., and LDH release was detected to measure cell death. **f** At 12 h after infection with *N. caninum* tachyzoites (MOI = 1:1 (**4**), 3:1 (**5**), 5:1 (**6**), parasite:cell), peritoneal macrophages with medium only (**1**), with 100 ng/ml LPS treatment (**2**), and 1 h after treatment with 5 mM ATP (**3**) were stained with a LIVE/DEAD Cell Imaging Kit for 15 min and imaged at a magnification of × 10. Live (green) and dead (red) cells in each group are shown. *Abbreviations*: C, control; SN, supernatants; LYS, cell lysates; MOI, multiplicity of infection. The data are representative of three independent experiments and presented as the mean ± SD (*P < 0.05; **P < 0.01; ***P < 0.001 *vs* negative control) *Scale-bars*: 50 μm

**Fig. 4:**
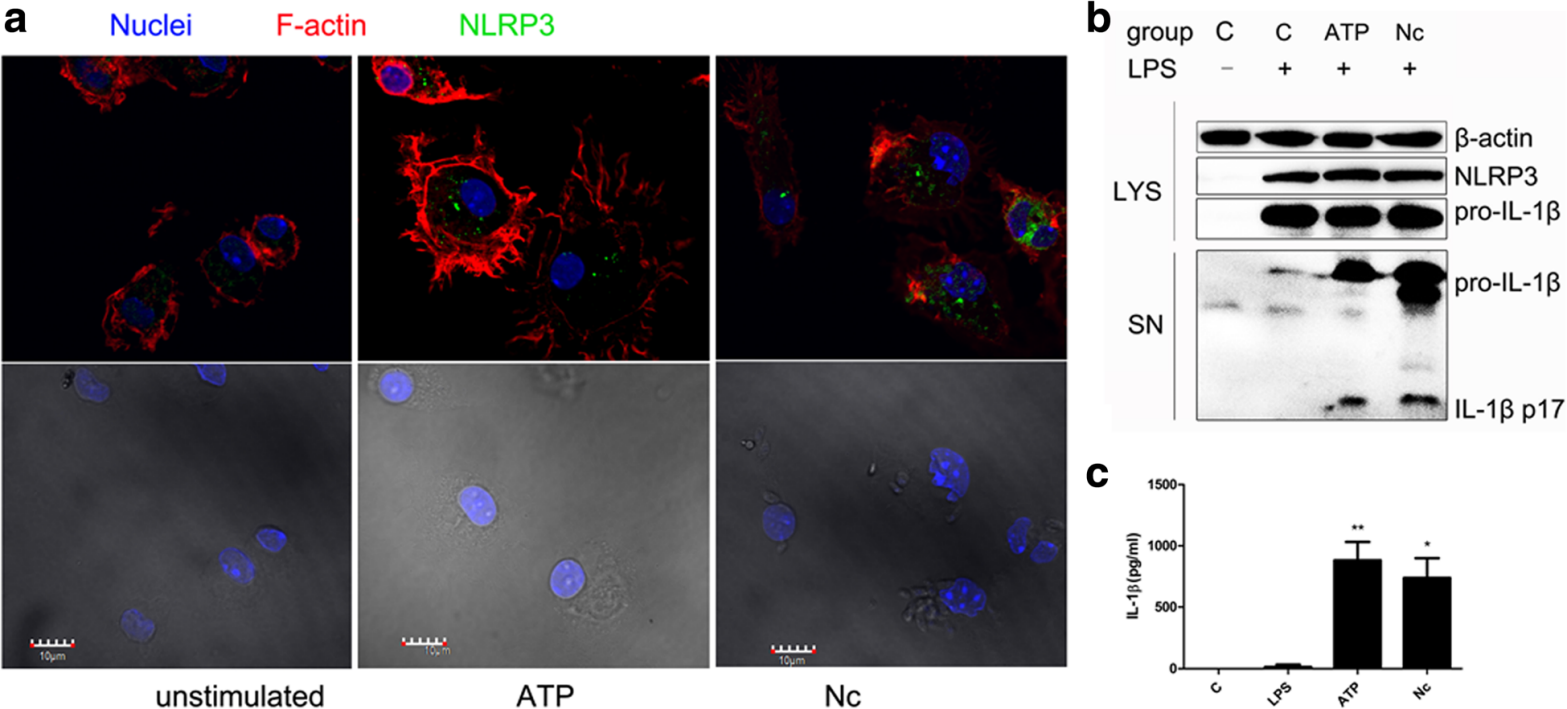
NLRP3 inflammasome activation in *N. caninum* infection. Peritoneal macrophages primed with LPS were incubated with *N. caninum* tachyzoites (*Nc*) (MOI = 3:1, parasite:cell) for 10 h or ATP (5 mM) for 30 min. **a** The localization of NLRP3 and F-actin after stimulation with *N. caninum* or ATP. **b** The NLRP3 expression and the secretion of IL-1β after stimulation with *N. caninum* (10 h) were determined by immunoblot analysis. **c** The production of IL-1β in the supernatants was measured by ELISA. *Abbreviations*: C, control; SN, supernatants; LYS, cell lysates; Nc, *N. caninum*. The data are representative of three independent experiments and presented as the mean ± SD (*P < 0.05; **P < 0.01; ***P < 0.001 *vs* negative control). *Scale-bars*: 10 μm

To further explore the inflammasome response to *N. caninum*, LPS-primed PMs were infected with different numbers of parasites. The cell death and distribution of NLRP3 in *N. caninum*-infected PMs were monitored. Further increases in IL-1β release at 12 h p.i. depended on incubation with higher number of parasites (MOI = 1:1, 3:1, and 5:1), and processed and secreted caspase-1 (caspase-1 p20) could also be detected in cell supernatants (Fig. 3a, b). Similarly, this phenomenon occurred in *N. caninum-*infected PMs at 3 h p.i. (Fig. 3c, d). Cell death was observed by both an LDH assay and a live/dead cell imaging assay at 12 h p.i. and was consistently dependent on the number of parasites used to infect the cells (Fig. 3e, f). In unstimulated macrophages, NLRP3 is localized throughout the cytoplasm, whereas in macrophages incubated with ATP, NLRP3 is redistributed to form puncta [14, 26]. Similarly, NLRP3 was localized to the perinuclear space in puncta and slightly distributed to the parasitophorous vacuole in PMs infected with *N. caninum* (3:1, parasite:cell) at 10 h p.i. (Fig. 4a). IL-1β release from *N. caninum-*infected PMs at 10 h p.i. was also observed (Fig. 4b, c), together with NLRP3 redistribution. These data indicated that NLRP3 inflammasome activation participated in the response to *N. caninum* infection.

### Role of the inflammasome in IL-1β release and control of *N. caninum* replication

**Fig. 5:**
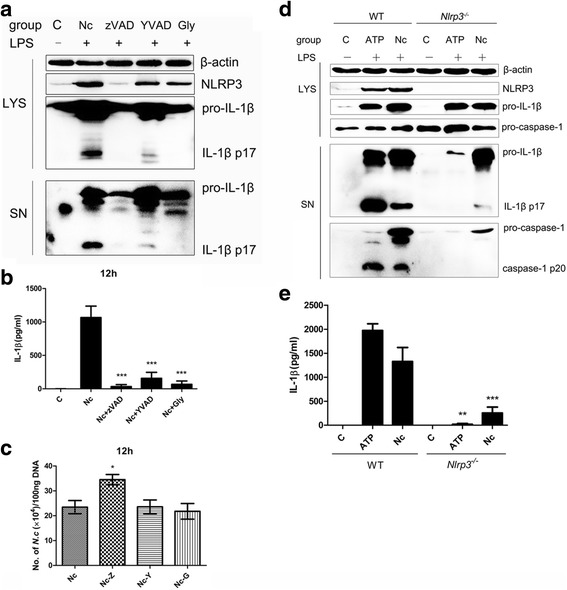
Role of inflammasomes in IL-1β release and control of *N. caninum* replication. Peritoneal macrophages were pre-treated with 100 μM zVAD-fmk (zVAD), 100 μM Ac-YVAD-CHO (YVAD), or 100 μM glyburide (Gly) before stimulation for 45 min, and after LPS priming for 3 h, the macrophages were infected with *N. caninum* (MOI = 3:1) for 12 h. **a** The NLRP3 expression and the secretion of IL-1β were determined by immunoblot analysis. **b** The production of IL-1β in the supernatants was measured by ELISA. **c** The number of *N. caninum* in infected macrophages was monitored after 12 h by qPCR. **d** With LPS priming wild-type (WT) and *Nlrp3* ^*−/−*^ peritoneal macrophages that were infected with *N. caninum* (MOI = 3:1) for 12 h, and NLRP3 expression, secretion of IL-1β and cleavage of caspase-1 were determined by immunoblot analysis. **e** The production of IL-1β in the supernatants of WT and *Nlrp3* ^*−/−*^ peritoneal macrophages stimulated with *N. caninum* was measured by ELISA. *Abbreviations*: C, control (with 0.2% DMSO in the inhibitory study); SN, supernatants; LYS, cell lysates; WT, wild-type; Nc, *N. caninum*; Z, zVAD-fmk; Y, Ac-YVAD-CHO; G, glyburide. The data are representative of three independent experiments and presented as the mean ± SD (*P < 0.05; **P < 0.01; ***P < 0.001 *vs* the Nc group or *Nlrp3* ^*−/−*^ group *vs* WT group)

To determine whether the inflammasome plays a role in IL-1β release and the control of parasite replication in macrophages infected by *N. caninum*, PMs were pre-treated with the pan-caspase inhibitor zVAD-fmk, the caspase-1-specific inhibitor Ac-YVAD-CHO or the NLRP3 inhibitor glyburide. Pan-caspase inhibition (zVAD) prevented the release of active IL-1β, and caspase-1 inhibition (YVAD) decreased the secretion of active IL-β. Although NLRP3 was expressed, glyburide inhibition led to failed IL-β maturation (Fig. 5a, b). For parasite replication in infected PMs, the parasite burden was assessed by qPCR with total DNA (100 ng) from infected cells. We observed that the parasite burden was significantly higher in the pan-caspase inhibition group than in the control group (Fig. 5c). Meanwhile, these inhibitors did not influence the viability of *N. caninum* at 12 h (see Additional file 1: Figure S1). These data indicate that the secretion of IL-1β in response to *N. caninum* is an inflammasome-mediated activity, and the inflammasome is required for the control of intracellular parasite replication.

To verify whether the NLRP3 inflammasome was required in host response to *N. caninum*, PMs isolated from *Nlrp3* ^*−/−*^ mice were infected with *N. caninum* (MOI = 3:1) at 12 h p.i. plus LPS priming. Results showed that release of active IL-1β in the supernatant was greatly reduced and cleavage of caspase-1 was inhibited in *Nlrp3* ^*−/−*^ PMs infected with *N. caninum*, compared to the amount present in the supernatant of wild-type (WT) infected PMs (Fig. 5d, e). Therefore, *N. caninum* induced IL-1β release and caspase-1 cleavage is mainly dependent on the NLRP3 inflammasome. These data confirmed the importance of NLRP3 inflammasome in *N. caninum* triggered inflammasome activation.

## Discussion

*Neospora caninum* is an intracellular protozoan parasite that causes severe economic losses in the dairy and beef industries. At present, aside from management control measures, no effective immunological approach is used to control neosporosis; therefore, understanding the innate immune recognition process for *N. caninum* will not only mechanistically define/improve the host resistance to this infectious disease but also help guide immune-mediated preventive approaches for *N. caninum* infection. In this study, we have utilized primary macrophages and employed inhibitory approaches to investigate whether the inflammasome was activated and its role in *N. caninum* infection. We found that macrophages responded to *N. caninum* infection with inflammasome activation and IL-1β secretion.

NLR inflammasome-related genes participated in the host response to *N. caninum*. Activation of the inflammasome requires a priming step, typically provided by LPS pre-treatment, which leads to the expression of sensor molecules and pro-forms of inflammatory cytokines (pro-IL-1β and pro-IL-18) through the NF-κB pathway [27]. In our study, the data demonstrated that several sensor molecules, especially NLRP3, were upregulated in PMs infected with *N. caninum* without LPS priming because the NF-κB pathway can be activated by *N. caninum* through TLR3 [5], TLR2 [9] and NOD2 [7]. The NLRP3 inflammasome senses and reacts to infection by numerous bacteria, viruses, fungi, and parasites, and this response can be protective for the host. With the activation of the inflammasome, activated caspase-1 proteolytically cleaves pro-IL1β and pro-IL-18 into active forms, and these secretions trigger pro-inflammatory and anti-microbial responses [11]. Macrophages express little or no pro-IL-1β, so IL-1β secretion includes two steps: pro-IL-1β induction and pro-IL-1β processing. The induction of pro-IL-1β is mediated through the transcriptional activation of the IL-1β gene in response to microbial stimulation via TLRs and NOD2 [8]. In *N. caninum-*infected PMs, IL-1β was extremely transcriptionally upregulated, and this step was the prerequisite for inflammasome activation [11]. Therefore, the upregulation of IL-1β and NLRP3 led us to explore the process of IL-1β secretion and NLRP3 inflammasome activation.

In addition, the IFNG (IFN-γ), IL 12B (IL-12p40), IL 12A (IL-12p35/a) and TNF (TNF-α) genes were extremely upregulated. Higher levels of IFN-γ and IL-12 are triggered in *N. caninum-*infected macrophages [28] and in *N. caninum*-infected mice [20], and they are critical cytokines for host resistance to *N. caninum* [29–32]. The production of TNF-α is also essential in controlling *N. caninum* infection [31, 33, 34]. In addition, similar to LPS, TNF-α is sufficient for NLRP3 inflammasome priming [35, 36].

A *T. gondii* study has shown a dual role for the inflammasome sensors NLRP1 and NRLP3 in murine resistance. In an in vitro study, the mRNA expression level and secretion of active IL-1β were observed in *T. gondii*-infected mouse bone marrow-derived macrophages (BMDMs), while neither upregulation nor cleavage of IL-18 were detected. However, in vivo experiments a high level of IL-18 production was observed in serum from *T. gondii*-infected mice, and paradoxically, no measurable IL-1β was detected in the serum [19]. The results of the above in vitro study of *T. gondii* infection were in agreement with our observation that the IL-18 gene was not upregulated. In addition, in future studies, we will focus on whether the phenomenon of IL-1β and IL-18 production observed in the *T. gondii* study occurs in *N. caninum*-infected mice.

Meanwhile, *T. gondii*-infected BMDMs from mice lacking *ASC, Nlrp1b*, *Nlrp3*, or both *Nlrp1b* and *Nlrp3* were used to determine the critical inflammasome components for IL-1β secretion, and the results showed that the secretion of IL-1β was highly related to the NLRP3 inflammasome. However, in addition to the essential role of NLRP3, the results from *T. gondii*-infected mice deficient in NLRP1 also revealed the protective role of NLRP1 in murine resistance to *T. gondii* [19]. In our study, NLRP3 was mostly upregulated without NLRP1a upregulation in *N. caninum*-infected PMs, and this result was similar to that observed in *T. gondii*-infected BMDMs. The NLRP3 inflammasome can sense various stimuli, such as bacteria, viruses, fungi, protozoa, extracellular ATP and uric acid crystals [13], while the activation of NLRP1 inflammasome is more restrictive, its best known agonist being anthrax lethal toxin [37]. However, the activation of both the NLRP3 and NLRP1 inflammasomes could induce IL-1β secretion and pyroptosis [13].

IL-1β secretion in *N. caninum-*infected PMs was time-dependent and more rapid and robust with LPS priming (LPS activates the NF-κB pathway). Meanwhile, macrophages responded to a higher number of *N. caninum* parasites with increased IL-1β secretion. It is possible that the IL-1β release mediated by the innate immune system could play a role in controlling *N. caninum* infection. Caspase-1-mediated cell death is called pyroptosis, is associated with the loss of plasma membrane integrity and release of DAMPs, and is a pro-inflammatory form of cell death [14] that mainly occurs in macrophages or dendritic cells [15, 38]. Following caspase-1 activation, *N. caninum*-infected peritoneal macrophages (with LPS priming) appeared to trigger cell death, with an increase in membrane permeability (live/dead cell image) and leakage of cytosolic contents (LDH release). The death of *N. caninum*-infected PMs may help the host eliminate the parasite because macrophages could transport *N. caninum* away from the primary infection site, and this process benefits parasite propagation in the host [39]. Results from studies in rats have shown that the rat NLRP1 sequence controls the macrophage sensitivity to pyroptosis in *T. gondii* infection and that the NLRP1 inflammasome correlates with macrophage cell death, parasite proliferation and IL-1β and IL-18 release [18, 40, 41], while pyroptosis does not occur in *T. gondii*-infected mouse BMDMs [19, 41]. In our study, LDH-related cell death is correlated with the MOI of *N. caninum*, while the confirmation of pyroptosis in *N. caninum-*infected PMs should be done by macrophages from *Caspase-1/11*- and *Gsdmd*-deficient mice because recent studies have found that GSDMD, an inflammatory caspase substrate, is the direct and final executor of pyroptosis. When cleaved by active caspase-1, the N-terminal fragment of GSDMD rapidly targets the membrane fraction of macrophages and induces the formation of plasma membrane pores, causing cell lysis [16, 17, 42]. In addition, *N. caninum* ESAs can trigger mild IL-1β release, and this release is independent of the concentration of ESAs, indicating its essential role in triggering the host response to *N. caninum* infection [39], but *N. caninum* ESAs failed to induce cell death (data not show). Therefore, live *N. caninum* played a dominant role in inflammasome activation.

The sensor NLRP3 was the most upregulated molecule, and we hypothesized that NLRP3 played a predominant role in sensing *N. caninum* infection. We observed the recruitment of NLRP3 into the perinuclear space and the formation of puncta, indicating the activation of NLRP3 inflammasome. And *N. caninum* infection in *Nlrp3* ^*−/−*^ PMs displayed greatly decreased IL-1β release and inhibited caspase-1 cleavage, this result revealed and confirmed that inflammasome-mediated secretion of IL-1β and cleavage of caspase-1 caused by *N. caninum* infection was highly dependent on NLRP3 inflammasome. The NLRP3 inflammasome is a critical component of innate immunity, which is thought to sense the disturbance of cellular homeostasis rather than directly recognize stimuli. Recently, the activation of the NLRP3 inflammasome in response to diverse stimuli has been proposed to be triggered by multiple cellular signals: K^+^ efflux, Ca^2+^ signaling, mitochondrial dysfunction, and lysosomal rupture [14, 43–45]. For example, extracellular ATP triggers the NLRP3 inflammasome through the cell surface receptor P2X7R and K^+^ efflux [14, 44]. The mechanism of NLRP3 inflammasome activation caused by *N. caninum* infection requires further study.

The inflammasome plays essential roles in restricting parasite replication. Caspase-1/ASC inflammasome components played a significant role in the control of *Trypanosoma cruzi* replication, but NLRP3 was shown to be dispensable in parasite survival [46]. NLRP3 inflammasome activation contributed to the restriction of *Leishmania* spp. replication in both in vitro and in vivo experiments [47]. *Toxoplasma gondii* infection in NLRP1-, NLRP3- and caspase-1/11-deficient mice showed that NLRP1, NLRP3 and caspase-1/11 were essential for the restriction of parasite loads and survival of mice [19]. In our study, when pre-treated with inhibitors, *N. caninum-*infected PMs showed an impaired induction of IL-1β and failed to restrict parasite replication. In addition, *N. caninum* infection in *Nlrp3* ^*−/−*^ PMs displayed extremely decreased release of IL-1β and inhibited cleavage of caspase-1. These data confirmed that the IL-1β release and caspase-1 cleavage were mediated by inflammasome activity in *N. caninum-*infected PMs, especially highly dependent on NLRP3 inflammasome. We speculate that the activation of the inflammasome plays an important role in controlling *N. caninum* infection. Future studies are required to determine the mechanism of inflammasome activation by *N. caninum* infection in inflammasome-deficient mice in vivo. In addition, it is intriguing that IL-1β secretion could be only partially decreased by a caspase-1 inhibitor, indicating that other caspases such as caspase-11 may participate in the inflammasome response to *N. caninum* infection, and further work will focus on this aspect.

## Conclusions

We first demonstrated that the NLRP3 inflammasome in macrophages can be activated by *N. caninum* infection and mediates caspase-1 processing and IL-1β release. In addition, the inflammasome plays an essential role in controlling *N. caninum* replication and survival in macrophages.

## Additional file

Additional file 1: Figure S1. Effect of inhibitors on *N. caninum* replication in Vero cells. To evaluate whether the inhibitors used in this study could influence *N. caninum* replication, the inhibitors zVAD-fmk (**z**), Ac-YVAD-CHO (**y**) and glyburide (**g**) were added to Vero cells for 45 min, and the Vero cells were then infected with *N. caninum* tachyzoites (MOI = 3:1; parasite:cell) for 12 h. Total DNA from the infected cells was extracted and was used for a quantitative analysis by qPCR described in this study. The results showed that only glyburide could slightly inhibit *N. caninum* replication (*P* = 0.0393), while zVAD-fmk and Ac-YVAD-CHO could not influence parasite replication compared with the Nc group. Meanwhile, parasitophorous vacuoles can be observed in each group (data not shown), indicating that these inhibitors did not influence the viability of *N. caninum*. (**P* <0.05; ***P* < 0.01; ****P* <0.001 *vs* the Nc group). (TIF 4400 kb)

## Abbreviations

ATP: Adenosine triphosphate
BSA: Bovine serum albumin
Ct: Threshold cycle
DAPI: 4’,6-diamidino-2-phenylindole
DMSO: Dimethyl sulfoxide
ESAs: Excretory secretory antigens
FBS: Fetal bovine serum
LDH: Lactate dehydrogenase
LPS: Lipopolysaccharide
MOI: Multiplicities of infection
NLRs: Nucleotide oligomerization domain (Nod)-like receptors
PBS: Phosphate buffer saline
PBST: Phosphate buffer saline with tween-20
PCR: Polymerase chain reaction
PMs: Peritoneal macrophages
qPCR: Quantitative polymerase chain reaction
SD: Standard deviation
TRITC: Tetramethylrhodamine isothiocyanate
