# Supplementary figures and images for: NLRP3 inflammasome activation in murine macrophages caused by Neospora caninum infection
Source: Parasit Vectors. 2017 May 30;10:266. doi: 10.1186/s13071-017-2197-2 (PMC5450200; doi:10.1186/s13071-017-2197-2)

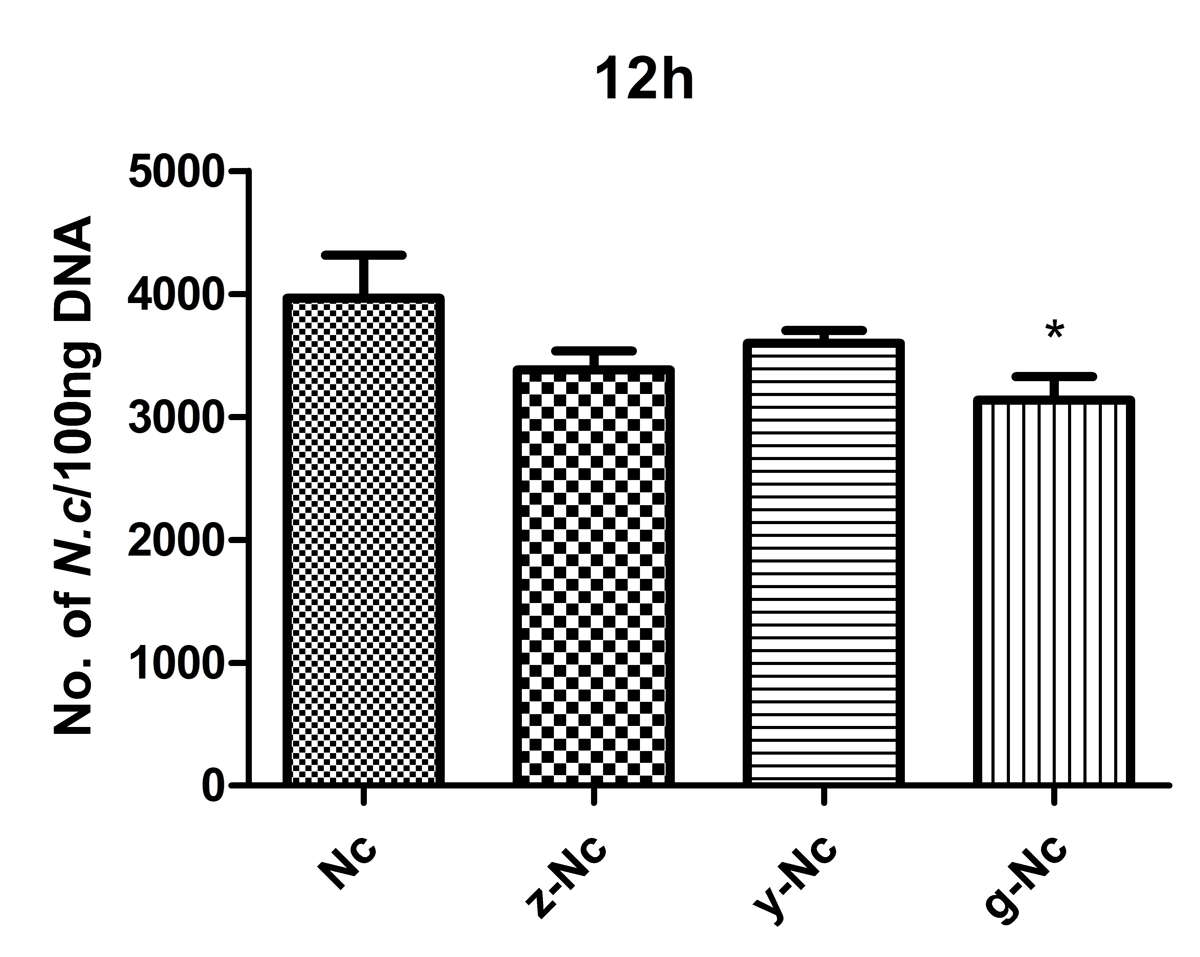

Supplement: Additional file 1: Figure S1. — Effect of inhibitors on N. caninum replication in Vero cells. To evaluate whether the inhibitors used in this study could influence N. caninum replication, the inhibitors zVAD-fmk (z), Ac-YVAD-CHO (y) and glyburide (g) were added to Vero cells for 45 min, and the Vero cells were then infected with N. caninum tachyzoites (MOI = 3:1; parasite:cell) for 12 h. Total DNA from the infected cells was extracted and was used for a quantitative analysis by qPCR described in this study. The results showed that only glyburide could slightly inhibit N. caninum replication (P = 0.0393), while zVAD-fmk and Ac-YVAD-CHO could not influence parasite replication compared with the Nc group. Meanwhile, parasitophorous vacuoles can be observed in each group (data not shown), indicating that these inhibitors did not influence the viability of N. caninum. (*P <0.05; **P < 0.01; ***P <0.001 vs the Nc group). (TIF 4400 kb) [file 13071_2017_2197_MOESM1_ESM.tif]
